# Validity and reliability of the Sinhalese version of the perceived stress scale questionnaire among Sri Lankans

**DOI:** 10.3389/fpsyg.2023.1152002

**Published:** 2023-06-16

**Authors:** Balapuwaduge Isuru Layan Madusanka Mendis, Palihaderu Arachchige Dineth Supasan Palihaderu, Panduka Karunanayake, Dilan Amila Satharasinghe, Jayasekara Mudiyanselage Krishanthi Jayarukshi Kumari Premarathne, Wajjakkara Kankanamlage Ruwin Rangeeth Dias, Iyanthimala Harshini Rajapakse, Avanti Sulochana Hapugalle, Wanasinghe Ranhettige Sasanka Anjalee Karunaratne, Agulugaha Gamage Yohan Nipuna Binendra, Kelaniya Bandaralage Pubudu Pradeep Kumara, Galathura Samanabaddage Dasun Prabhashwara, Upul Senarath, Swee Keong Yeap, Wan Yong Ho, Arosha Sampath Dissanayake

**Affiliations:** ^1^Department of Basic Veterinary Sciences, Faculty of Veterinary Medicine and Animal Science, University of Peradeniya, Peradeniya, Sri Lanka; ^2^Department of Clinical Medicine, Faculty of Medicine, University of Colombo, Colombo, Sri Lanka; ^3^Department of Livestock and Avian Sciences, Faculty of Livestock, Fisheries and Nutrition, Wayamba University of Sri Lanka, Makandura, Gonawila, Sri Lanka; ^4^Department of North Indian Music, Faculty of Music, University of the Visual and Performing Arts, Colombo, Sri Lanka; ^5^Department of Psychiatry, Faculty of Medicine, University of Ruhuna, Galle, Sri Lanka; ^6^Department of Community Medicine, Faculty of Medicine, University of Colombo, Colombo, Sri Lanka; ^7^China-ASEAN College of Marine Sciences, Xiamen University Malaysia, Sepang, Selangor, Malaysia; ^8^Division of Biomedical Sciences, Faculty of Science and Engineering, University of Nottingham Malaysia, Semenyih, Malaysia; ^9^Department of Clinical Medicine, Faculty of Medicine, University of Ruhuna, Galle, Sri Lanka

**Keywords:** perceived stress, Sri Lanka, validation, psychometric, Sinhala, Type 2 Diabetes Mellitus

## Abstract

**Introduction:**

Despite the availability of validated psychometrics tools to assess depression, there has not been any validated and reliable tool established to test perceived stress among Sri Lankans. The objective of this study is to test the validity and reliability of the Sinhalese Version of the Sheldon Cohen Perceived Stress Scale.

**Materials and methods:**

Standard and systematic procedures were adopted to translate the original English version of the Perceived Stress Scale-10 questionnaire into Sinhalese. Consecutive sampling was employed to recruit the Type 2 Diabetes mellitus (T2DM) sample (*n* = 321), and a convenient sampling was used to recruit the Age and Sex matched Healthy Controls (ASMHC) (*n* = 101) and the Healthy Community Controls (HCC) groups (*n* = 75). Cronbach alpha was used to assess internal consistency and reliability was determined using test–retest method utilizing Spearman’s correlation coefficient. Sensitivity was evaluated by comparing the mean scores of the Sinhalese Perceived Stress Scale (S-PSS-10) and Sinhalese Patient Health Questionnaire (S-PHQ-9) scores. *Post-hoc* comparisons were done using Bonferroni’s method. Mean scores were compared between the T2DM, ASMHC, and HCC groups using the independent *t*-test. Explanatory Factor Analysis (EFA) was conducted using the principal component and Varimax rotation while the Confirmatory Factor Analysis (CFA) was performed to assess the goodness-of-fit of the factor structure extracted from the EFA. Concurrent validity was assessed using the Pearson correlation between the S-PSS-10 and Patient Health Questionnaire measured by S-PHQ-9 (*p* < 0.05).

**Results:**

Cronbach alpha values of the three groups T2DM, ASMHC and HCC were 0.85, 0.81, and 0.79, respectively. Results of the ANOVA test suggested that there was a significant difference in the mean scores between groups (*p* < 0.00). EFA analysis revealed the existence of two factors with eigenvalues greater than 1.0. The factor loadings for the items ranged from 0.71–0.83. The CFA analysis demonstrated a good model fit for the two-factor model S-PSS-10. The S-PSS-10 significantly correlated with S-PHQ-9, indicating an acceptable concurrent validity.

**Conclusion:**

Findings revealed that the S-PSS-10 questionnaire can be used to screen perceived stress among the majority of the Sri Lankan Sinhalese-speaking population specially with chronic illnesses. Further studies with higher sample sizes across different populations would enhance the validity and reliability of S-PSS-10.

## Introduction

Psychological stress is increasingly being recognized as a risk factor for the onset and progression of non-communicable diseases (Pothisiri et al., 2022; Gebicki et al., 2023). The normal physiological response to any form of stress is adaptive, enabling the organism to withstand the stress and maintain body homeostasis. “Allostasis” is a mechanism which helps the body to adapt to various stressors and involves cerebral neurohumoral mechanisms (Schneiderman et al., 2005; Parker et al., 2022). However, when exposed to chronic stress which results in prolonged stimulation of the allostatic system, the adaptive mechanisms fail. This is termed “Allostatic load,” and involves dysregulation of multiple neuroendocrine, cardiovascular, metabolic and inflammatory pathways (Goymann and Wingfield, 2004; Carbone, 2021; Guidi et al., 2021).

Despite the inherent difficulties to assess stress with the composite psychological, social, and biological variables, different methods have been developed to quantify stress. For example, a clinical criteria and diagnostic interviews are established to quantify the allostatic load (Fava et al., 2019). Also, several types of biomarkers in serum can be used to assess the impact and magnitude of stress (i.e., cortisol, adrenocorticotropic hormone, cytokine profiles) (Kokka et al., 2022; Li et al., 2023). In addition, there are many self-assessment questionnaires, scales, and tests developed to assess psychometrics like perceived stress, anxiety and depression (i.e., Perceived Stress Scale, Patient Health Questionnaire- 9, Depression and Anxiety Scale, Ardell Wellness Stress Test) (Cohen et al., 1983; Peacock and Wong, 1990; Oei et al., 2013). For the issue at hand specifically, the perceived stress levels can be taken as the measure as it contributes in multiple ways to their level of awareness, coping and ability to self-manage the disease. Perceived stress refers to the “degree to which events in a person’s life are assessed as stressful, unpredictable and uncontrollable.” The importance of Perceived Stress is that it encompasses multiple dimensions, including awareness, coping and the ability to self-manage (González-Ramírez et al., 2013). Several rating scales, such as stress appraisal measure, perceived stress scale and impact of event scale have been developed to assess perceived stress (Peacock and Wong, 1990; Feizi et al., 2012).

Among them, the Sheldon Cohen Perceives Stress Scale is the most widely adopted and used scale among these psychometric instruments (Lee et al., 2015; Gamonal-Limcaoco et al., 2021; Mozumder, 2022). It is an instrument that measures the level of perception of stress concerning unpredictability, lack of control, and overload. The 10-item Perceived Stress Scale (PSS) questionnaire encompasses the two-factor structure, assessing both the “negative feelings and the inability to handle stress” as well as “positive emotions and the ability to take action in stressful situations” (Cohen et al., 1983; Lee, 2012). A 5-point Likert scale indicates how often a participant experienced a particular emotion or thought over the past 4 weeks. Higher scores correspond with higher levels of perceived stress. Notably, it has been reported that the PSS-10 correlated with other psychosocial psychometrics like depression, anxiety and low self-esteem (Roberti et al., 2006; Wang et al., 2022).

As a result of the exceptional psychometric properties, the PSS-10 has been translated and culturally adapted for use in different languages including Arabic, Hindi, Chinese, Mexican, Greek, Swedish and Malaysian and across various populations with diabetics, smokers, pregnant and postpartum women, emphasizing the importance of validating this tool across various populations (Chaaya et al., 2010; Lee, 2012). For adult populations, validated normative data is available in various countries like Germany, Greece, Bangladesh, Mexico and Sweden. According to the literature, the psychometric properties for the validity of PSS-10 are assessed mainly using internal consistency, test–retest reliability and construct validity, concurrent validity (see Table 1). Internal consistency refers to how much the similar items in the scale have correlated with each other and it is assessed using Cronbach’s alpha (Hayes and Coutts, 2020). Test–retest reliability indicates the extent to which the same subjects have responded similarly to the questionnaire during a fleeting time period and a statistical correlation of the PSS-10 scores between the two time points of the same subjects are compared to assess the outcome (Noble et al., 2019).

**Table 1:** Validity and reliability of PSS-10 with other languages.

Language and Reference	Sample description	Internal consistency (Cronbach’s alpha)	Reliability (test–retest)	Concurrent validity (*r* = correlation coefficient)	Construct validity							Sensitivity analysis
					EFA (PCA)	CFA (χ^2^/df)	*p*-value	RMSEA	NFI	CFI	TLI	
Bengali (Mozumder, 2022)	Adults between 18 and 64 years from eight divisional districts (*N* = 315)	0.71	*r* = 0.74, *p* < 0.01	With GHQ-28 (*r* = 0.57, *p* < 0.01)	N/A	In the modified model = 1.95	0.00	0.05	N/A	0.95	0.93	Yes. With the two groups
Spanish (Remor, 2006)	Participants (*N* = 440) with different medical conditions (18–69 years)	0.82	*r* = 0.77, *p* < 0.00	With HADS-T/distress (*r* = 0.72) and HADS-A/anxiety (*r* = 0.66, *p* < 0.001)	N/A	N/A	N/A	N/A	N/A	N/A	N/A	Yes. With four groups
Mexican (González-Ramírez et al., 2013)	Participants (*N* = 1990) mean age of 35.5 ± 13.3 years old	Men (0.77) and women (0.78)	N/A	N/A	Yes, two factors identified	Gender group = 5.03	Not given	0.045	0.95	0.96	0.94	No. Compared with gender and age groups.
						Age group = 3.23	Not given	0.034	0.88	0.92	0.92	
Chinese (Liu et al., 2020)	Chinese senior high school students (*N* = 1574) and mean age of 15.26 ± 0.56 years	0.79	N/A	With SCARED (*r* = 0.43) and DSRSC (*r* = 0.42) (*p* < 0.001)	N/A. two factor model used	χ^2^ (34) = 332.22 only	<0.001	0.075	0.90	0.92	N/A	No. Compared invariances with gender groups
Arabic (Chaaya et al., 2010)	A total of 268 women (113 pregnant, 97 postpartum and 58 healthy females) and mean age 27.6 ± 5.5 years	0.74	*r* = 0.74, *p* < 0.05	With GHQ-12 (*r* = 0.59) and EPDS (*r* = 0.49) (*p* < 0.05)	Yes, PCA resulted 2 factors	N/A	N/A	N/A	N/A	N/A	N/A	Yes. With three groups
Korean (Lee et al., 2015)	A total of 402 people with chronic diseases and mean age 58.56 ± 12.91 years	0.75	*r* = 0.81, *p* < 0.05	With CES-D scale (*r* = 0.66, *p* < 0.001)	Yes, PCA resulted 2 factors	2.46	GFI = 0.92	0.08 (0.06–0.10)	0.88	0.93	N/A	No. Compared with gender and effect sizes
Theligu (Tikka et al., 2022)	A total of 311 healthcare workers and mean age 38.35 ± 7.85 years	0.75–0.71 and SHR = 0.8	N/A	GAD-7 (*r* = 0.54) and PHQ-9 (*r* = 0.45) (*p* < 0.001)	Yes, PCA extracted 2 factors	N/A	N/A	N/A	N/A	N/A	N/A	N/A
Amharic (Tsegaye et al., 2022)	A total of 758 undergraduate students and mean age 26.3 ± 5.8 years	0.77	N/A	N/A	Yes, PCA resulted 2 factors	1.9	<0.001	0.04 (0.03–0.06)	N/A	0.96	N/A	N/A
Italian (Mondo et al., 2021)	A total of 649 precarious workers and mean age 39.6 ± 10.1 years	0.75	N/A	With BDI-II (*r* = 0.45, *p* < 0.001)	Yes, extracted two factors	2.5	N/A	0.07 (0.05–0.09)	N/A	0.95	0.93	N/A
Greek (Andreou et al., 2011)	A total of 941 individuals and mean age 29 years	0.82	N/A	With DASS-21 for stress (*r* = 0.644), depression (*r* = 0.606), and anxiety (*r* = 0.542) (*p* < 0.001)	No, two factor model used	4.86	<0.001	0.065	N/A	0.94	N/A	N/A

N/A, Not Assigned; PCA, Principal Component Analysis; EFA, Exploratory Factor Analysis; CFA, Confirmatory Factor Analysis; RMSEA, Root Mean square Error of Approximation; NFI, Normed Fit Index; CFI, Comparative Fit Index; TLI, Tucker-Lewis Index; GHQ-28, General Health Questionnaire-28; HADS, Hospital Anxiety and Depression Scale; DSRSC, Depression Self-Rating Scale for Children; SCARED, Screen for Child Anxiety Related Emotional Disorders; CES-D, Center for Epidemiologic Studies Depression Scale; SHR, Split half reliability; GAD-7, Generalized Anxiety Disorder-7; BDI-II, Beck Depression Inventory, 2nd Edition; DASS-21, Depression Anxiety and Stress Scale-21.

Even though the PSS-10 is a unidimensional indicator of perceived stress, validation studies have revealed the existence of commonly reported two-factors namely ‘perceived helplessness’ and ‘perceived self-efficacy’ (Liu et al., 2020; Tikka et al., 2022). To assess the existence of the factor structure and validate the construct of questionnaires, factor analysis statistical techniques can be used (i.e., EFA and CFA). The EFA analysis is used to identify these two factors (theoretical constructs) and underlying factor structure. Although the EFA provides evidence for the two-factor structure of the PSS-10, it cannot measure the variables. The verification of the two constructs of the PSS-10 is carried out using the CFA statistical technique and it facilitates the researcher to test the hypothesis between observed PSS-10 variables and underlying two latent constructs. In general, the EFA is used to determine the factor structure of the PSS-10 while CFA provides the verification for the two-factor structure (Suhr, 2006). The concurrent validity is conducted using a correlation analysis with a similar validated tool which assesses stress or stress-related parameter. On the other hand, convergent validity explore how two theoretically related constructs are actually related and it can be executed with a correlation analysis with similar stress related construct (i.e., stressful life events).

However, the PSS-10 has not been translated into Sinhalese or validated for use in the Sri Lankan population including those with diabetes. The main language that the Sri Lankans speak is Sinhalese, who make up about 75% of the country’s population. Majority of the other ethnicities residing in Sri Lanka also can communicate in Sinhalese. Considering, the importance and necessity of the locally translated and validated perceived stress assessment questionnaire, we have made an attempt to translate and validate the PSS-10 into Sinhalese for wider use. The hypothesis of our study is that the S-PSS-10 would also exhibit a two-factor structure during the EFA and demonstrate satisfactory reliability and validity scores during the Confirmatory factor analysis as well. Also, we expect a positive statistically significant correlation with a complimentary stress assessment questionnaire to support the concurrent validity of the tool. Therefore, the primary outcome of this study is to assess the validity and reliability of a newly translated S-PSS-10 in a Sri Lankan population able to communicate the Sinhalese language, which is culturally appropriate and practical to use in a busy and resource-limited clinic setting, both for research purposes and also to be used in the clinical management of such patients.

## Materials and methods

### Participants

There were three groups in the study population (*n* = 497). These included previously diagnosed Type 2 diabetes mellitus (T2DM) patients (*n* = 321), their age and sex matched healthy control (ASMHC) sample (*n* = 101) and healthy community controls sample (HCC) residing in urban and suburban areas of Sri Lanka who were participants in the pilot study (*n* = 75). The determination of minimum sample size was assessed at 0.9 power and 0.05 alpha (the probability of type I error) values based on the prevalence of perceived stress in prior conducted studies (Bernard, 2011). The incidence of perceived stress among type 2 diabetes was reported between 39.3–50% (Mortensen et al., 2022; Vidyulatha et al., 2022). Hence, we have taken 40% anticipated incidence of perceived stress among T2DM population and 50% in our study group.


$$\begin{array}{c} N=\frac{p_{0}q_{0}{\left\{z_{1-\alpha /2}+z_{1-\beta }\sqrt{\frac{p_{1}q_{1}}{p_{0}q_{0}}}\right\}}^{2}}{{\left(p_{1}-p_{0}\right)}^{2}}\\ q_{0}=1-p_{0}\\ q_{1}=1-p_{1}\\ N=\frac{0.4*0.6{\left\{1.96+1.28\sqrt{\frac{0.5*0.5}{0.4*0.6}}\right\}}^{2}}{{\left(0.5-0.4\right)}^{2}}\\ N=256 \end{array}$$

*p*_0_ = proportion (incidence) of population

*p*_1_ = proportion (incidence) of study group

*N* = sample size for study group

*α* = probability of type I error (0.05)

*β* = probability of type II error (0.1)

*z* = critical Z value for a given α or β

Also, the prevalence of perceived stress and depression among people in Sri Lanka and low- middle income countries were reported as 6.2 and 6.1% (Cristóbal-Narváez et al., 2020). The prevalence of perceived stress among elderly was recorded as 27.8% in Sri Lanka (Malhotra et al., 2010). Hence, we anticipated a 6.1% prevalence in the HCC group and 27.8% in ASMHC group.


$$\begin{array}{c} {\left.\begin{array}{l} N_{1}=\{z_{1-\alpha /2}*\sqrt{\bar{p}*\bar{q}*\left(1+\frac{1}{k}\right.})+z_{1-\beta }*\\ \;\;\;\;\;\;\;\;\;\;\;\;\;\;\;\;\;\;\sqrt{p_{1}*q_{1}+\left(\frac{p_{2}*q_{2}}{k}\right)} \end{array}\right\}}^{2}/\Delta^{2}\\ q_{1}=1-p_{1}\\ q_{2}=1-p_{2}\\ \bar{p}=\frac{p_{1}+kp_{2}}{1+K}\\ \bar{q}=1-\bar{p}\\ {\left.\left.\begin{array}{l} N_{1}=\{1.96*\sqrt{0.1695*0.8305*\left(1+\frac{1}{1}\right.})+1.28*\\ \;\;\;\;\;\;\;\;\;\;\;\;\;\;\;\;\;\;\;\;\;\;\;\;\;\;\;\sqrt{0.061*0.939+\left(\frac{0.278*0.722}{1}\right.} \end{array}\right)\right\}}^{2}/0.217^{2}\\ N_{1}=61\\ N_{2}=K*N_{1}=61 \end{array}$$

*p*_1_, *p*_2_ = proportion (incidence) of group 1 and 2

Δ = |*p*_2_–*p*_1_| = absolute difference between two proportions

*n*_1_ = sample size for group 1

*n*_2_ = sample size for group 2

*α* = probability of type I error (0.05)

*β* = probability of type II error (0.1)

*z* = critical *Z* value for a given α or β

*K* = ratio of sample size for group 2 to group 1

**Figure 1:**
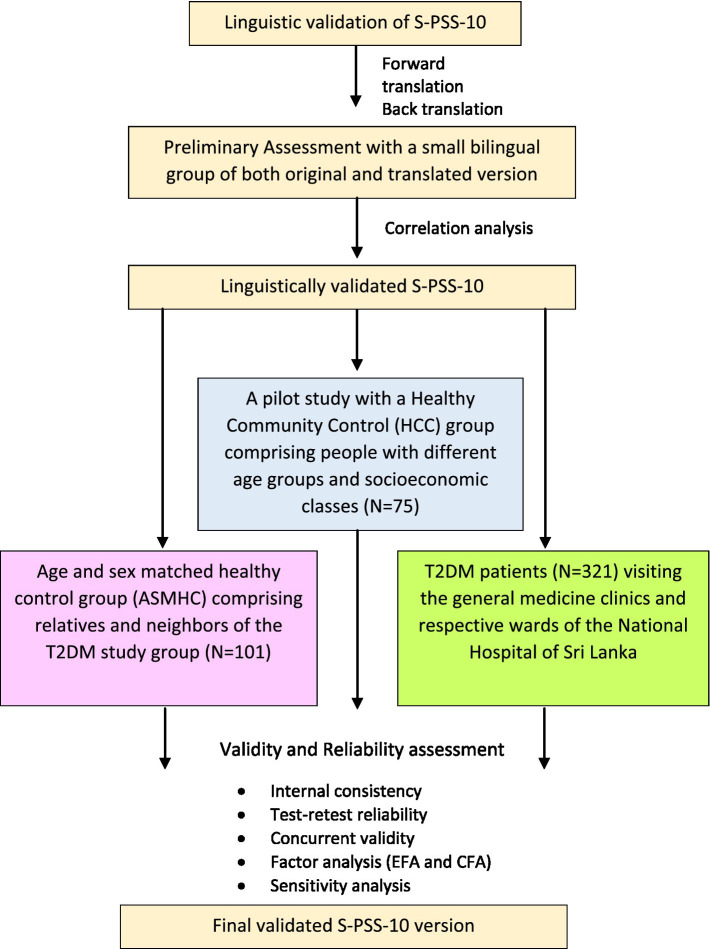
Flow chart of the study.

The T2DM patients were recruited from the general medicine clinic and its respective wards of the National Hospital of Sri Lanka (NHSL) over a four-month period. The majority of patients with T2DM in Sri Lanka receive treatment at general medical clinics conducted in the outpatient departments of hospitals. The NHSL, in particular, receives patients from all provinces of the country’s tertiary care hospital. Thus, the patient population in this study can be considered comparable to the diabetic patient population in Sri Lanka. Consecutive patients were approached and those who provided consent for the study were included in the T2DM group. Both the World Health Organization (WHO) and American Diabetes Association Guidelines (ADA) were used as the basis for diagnosing T2DM patients (World Health Organization and International Diabetes Federation, 2006; American Diabetes Association, 2020). Patients with fasting plasma glucose levels above ≥126 mg/dL (7.0 mmol/L) and HbA1c levels above ≥6.5% (48 mmol/mol) were included in the study. Also, patients with severe psychiatric disorders and severe visual and hearing impairments were excluded. Convenient sampling technique was used to recruit both the ASMHC and HCC control groups. Most of the subjects in the ASMHC group were relatives and neighbors of the T2DM study group and people residing close to the NHSL. The flow chart of the study is presented in the Figure 1. The HCC group comprised participants representing the age groups and economic classes residing in Kalutara, Kandy, Kurunegala, Galle, and Colombo districts (see Table 2).

**Table 2:** Study design of the healthy community control group (HCC).

Social classes	Age 23–38 (Millennials or Gen-Y)	Age 39–54 (Gen-X)	55–73 (Baby Boomers)	Total
Upper-class	5	5	5	15
Upper-middle class	5	5	5	15
Middle class	5	5	5	15
Working class	5	5	5	15
Lower class	5	5	5	15
Total	25	25	25	75

#### Instruments

The Sheldon Cohen 10-item Perceived Stress Scale was used to assess the perception of stress. The questionnaire is structured on a five-point scale (0-never, 1-almost never, 2-sometimes, 3-fairly often, 4-very often) which the participants are required to quantitatively respond to their thoughts and feelings with regard to life events and situations during the preceding month. The general scoring procedure was adopted for questions 1, 2, 3, 6, 9, and 10 where the rated score from the scale of the respective questions was directly summated. The same reverse scoring procedure was followed for questions 4, 5, 7, and 8. Each 10 items’ scores were summated to obtain a final score and were used to calculate the perceived stress levels. Perceived stress scores between 0 and 13 were considered ‘Low,’ 14–26 ‘Moderate’ and 27–40 ‘High’ Perceived Stress Levels (Cohen et al., 1983).

#### Linguistic validation

The standard and systematic procedure with the cross-cultural translation guidelines was followed to translate the original English version of the PSS-10 questionnaire into Sinhalese (Bullinger et al., 1998; DeVotta, 2016). Two professional translators of the Sinhalese language conducted the forward translation. After the translation, a bilingual psychiatrist and consultant physician who were not familiar with the scale reviewed for the appropriateness of the Sinhalese version. The reconciled version of the forward translated questionnaire was also assessed for its’ similarity with the original PSS-10. The reviewed version was back-translated by another two independent professional translators who have not seen the original questionnaire. To ensure consistency with the original PSS, the disparities were addressed accordingly. The back-translated version was piloted among 15 university students to verify that the questionnaire was clear and comprehensible. The final review and the corrected version of the questionnaire were done by the project investigators through cognitive debriefing. Subsequently, a separate group of students (bilingual) were asked to complete the Sinhalese and English version of the PSS-10 (*n* = 20) in two rounds. The average time between the two administrations was 10 min. The Spearman correlation coefficient between the Sinhalese and English versions was reported as 0.7 (*p* < 0.05) and demonstrated an acceptable level of linguistic validity. These 20 students were not included in the final study sample.

#### Reliability

Reliability of the Sinhalese PSS-10 (S-PSS-10) was assessed using a test–retest procedure conducted among the T2DM and ASMHC groups. The 40 respondents who participated in the T2DM group (after 3–4 weeks during their next clinic day) and 60 subjects from the ASMHC group were randomly selected and invited for a retest after 15 min. The response rate of the retest procedure was recorded as 88.3% (53 subjects).

#### Administration

A medical officer and two trained research assistants approached the subjects. Information about the study was provided for all using an information sheet. The queries and clarifications were answered verbally upon their request. Subsequently, informed written consent from the participants was obtained. The T2DM study group subjects answered both the self-administered S-PSS-10 and S-PHQ-9 including a structured questionnaire (demographics and medical history) and with the help of the medical officer and research assistants. Similarly, the questionnaire was self-administered by the healthy control group and community control groups. Research assistants were available for any clarification. For the test–retest, the S-PSS-10 was provided twice to the T2DM patients (*n* = 40) and ASMHC group subjects (*n* = 53) for the reliability assessment.

#### Ethical considerations

The ethical clearance was obtained from the Ethics Review Committee (ERC), Faculty of Medicine, University of Colombo Sri Lanka under reference numbers EC-20-010 and EC-19-106. The study was also registered under the Sri Lanka clinical trials registry under the registration number SLCTR/2021/012. The PHQ-9 and PSS-10 make a sensitive inquiry into the mental state of patients and indicate at times on the presence of anxiety/depression. Patients who had high scores for depression were referred to the ward and clinic medical officers for further evaluation.

### Data analysis

#### Descriptive statistics of the participants

Descriptive statistics of the three groups were reported as means and standard deviations. The distribution of respondents’ perceived stress levels and level of depression according to the categorization of the PSS-10 and PHQ-9, respectively have been represented using percentage values.

#### Internal consistency and reliability

Statistical analyses were performed with SPSS version 26.0 (SPSS Inc., Chicago, IL, United States). Internal consistency was measured using Cronbach’s alpha coefficient for each sample. The test–retest reliability of the S-PSS-10 was assessed using Spearman’s correlation coefficient (rho).

#### Sensitivity analysis

The one-way ANOVA compared the mean S-PSS-10 and S-PHQ-9 scores between the three groups. Post-hoc comparisons were done using Bonferroni’s method. Mean scores were compared between the T2DM, ASMHC, and HCC groups using the independent *t*-test.

#### Construct validity

Explanatory Factor Analysis (EFA) was conducted using the principal component and Varimax rotation with Kaiser normalization. The sample adequacy was assessed by the Kaiser-Meyer-Olkin (KMO) measure of sampling adequacy. Confirmatory Factor Analysis (CFA) was performed to assess the goodness-of-fit of the factor structure extracted from the EFA. The CFA analyses were conducted using IBM SPSS Statistics AMOS version 23.0 (SPSS Inc., Chicago, IL, United States). The maximum-likelihood estimation (MLE) method was employed to test the covariance matrix to assess the model fit. Furthermore, the structural equation modeling (SEM) in AMOS was also conducted to assess the model fit using path diagrams and standardized regression weights. All the other analyses were performed with SPSS version 26.0 (SPSS Inc., Chicago, IL, United States). The *p*-values less than *p* < 0.05 were considered significant.

#### Concurrent validity

The concurrent validity of the PSS-10 questionnaire was assessed by administering a validated Sinhalese Patient Health Questionnaire (S-PHQ-9) for both groups concurrently (Hanwella et al., 2014). The PHQ-9 is an instrument to monitor the severity of depression and response to treatment. The questionnaire possesses nine questions, based on the gold standard DSM-IV criteria of depression. The sensitivity and specificity of the PHQ-9 tool were previously reported as 0.75 and 0.97, respectively. As per the literature, the PSS-10 and PHQ-9 have been observed to correlate positively As the T2DM patients have chronic illnesses and comorbidities, we expected higher stress levels among this population when compared with the healthy control group. Hence, we anticipated a positive association between the two tools. Evidence of concurrent validity was assessed by Pearson correlation between the S-PSS-10 and nine-item Patient Health Questionnaire measured by S-PHQ-9, respectively (*p* < 0.05).

## Results

### Descriptive statistics of the participants

The mean age of the three samples T2DM, ASMHC and HCC were 58.4 ± 10.2, 57.0 ± 11.2, and 46.2 ± 13.8, respectively. The majority among the T2DM and its age and sex match healthy control group were females with 62 and 60% percentages, while there were 56% males in the healthy community control group. The percentage distributions of the perceived stress levels calculated according to the respective scores across the three groups were T2DM (L-5%, M-53%, and H-42%), age and sex matched healthy control (L-15%, M-50%, and H-35%) and community controls (L-8%, M-56%, and H-36%). There were 10% with moderate depression in T2DM group, whereas only 2% found in the ASMHC group (see Table 3).

**Table 3:** Demographic and psychometric characteristics of the participants.

Description	Total	T2DM patients	Age and sex matched healthy controls	Healthy community controls
Sample size	497	321	101	75
Age	56.3 ± 11.8	58.4 ± 10.2	57.0 ± 11.2	46.2 ± 13.8
Gender	Male 43% Female 57%	Male 38% Female 62%	Male 47% Female 53%	Male 56% Female 44%
Perceived stress levels (S-PSS-10)	Low (L) 14% Moderate (M)56% High (H) 30%	Low (L) 5% Moderate (M) 54% High (H) 41%	Low (L) 15% Moderate (M) 59% High (H) 26%	36% Low (L) 56% Moderate (M) 8% High (H)
Severity of depression and response to treatments (S-PHQ-9)	–	Normal 26% Mild 45% Moderate 22% Moderate–severe 6% Severe 1%	Normal 69% Mild 18% Moderate 12% Moderate–severe 1% Severe 0%	–

### Internal consistency and reliability

The mean perceived stress scores among the groups were reported as total: 21.4 ± 6.9, T2DM: 23.9 ± 6.0, ASMHC: 17.9 ± 6.0, and HCC:15.8 ± 6.4. All Cronbach’s alpha values of the three groups were reported above 0.7 indicating a good internal consistency reliability of the S-PSS-10. The Cronbach’s alpha values ranged from 0.79 to 0.87. The mean scores of the S-PSS-10 item scale comprising the two subscales and corresponding standard deviations are presented in Table 4. The Spearman correlation coefficient value for the test–retest was obtained as 0.74 and 0.92 in T2DM and ASMHC groups, respectively and the correlation was significant at the 0.01 level (two-tailed).

**Table 4:** Mean scores, standard deviations and internal consistency of the Sinhalese S-PSS-10.

Description	Total	T2DM patients	ASMHC group	HCC group	*P* value (ANOVA)
Mean scores	21.4 ± 6.9	23.8 ± 6.0	17.9 ± 6.0	15.8 ± 6.4	0.00
Perceived distress	13.7 ± 4.8	15.0 ± 4.2	11.6 ± 4.9	11.2 ± 5.2	
PSS-Q1	2.4 ± 0.9	2.6 ± 0.8	2.0 ± 1.1	2.0 ± 0.9	
PSS-Q2	2.2 ± 1.0	2.4 ± 0.9	1.7 ± 1.0	1.6 ± 1.2	
PSS-Q3	2.2 ± 1.0	2.4 ± 0.9	1.9 ± 1.0	1.8 ± 1.2	
PSS-Q6	2.2 ± 0.9	2.4 ± 0.8	2.0 ± 0.9	1.8 ± 1.2	
PSS-Q9	2.3 ± 0.9	2.5 ± 0.8	2.0 ± 0.9	2.0 ± 0.9	
PSS-Q10	2.2 ± 1.0	2.4 ± 0.9	1.8 ± 0.9	1.8 ± 1.1	
Perceived coping	7.6 ± 3.4	8.8 ± 3.0	6.3 ± 3.0	4.6 ± 2.9	
PSS-Q4	1.8 ± 1.0	2.2 ± 0.9	1.4 ± 0.8	0.9 ± 0.9	
PSS-Q5	2.0 ± 0.9	2.2 ± 0.8	1.6 ± 0.8	1.3 ± 0.9	
PSS-Q7	1.9 ± 1.1	2.1 ± 1.1	1.7 ± 1.0	1.2 ± 1.0	
PSS-Q8	1.8 ± 1.0	2.1 ± 0.9	1.5 ± 0.9	1.1 ± 1.0	
Cronbach’s alpha for PSS-10	0.87	0.85	0.81	0.79	
Cronbach’s alpha for perceived distress	0.89	0.87	0.90	0.86	
Cronbach’s alpha for perceived coping	0.83	0.79	0.82	0.73	
Mean scores S-PHQ-9		7.35 ± 4.23	3.76 ± 3.80		
Cronbach’s alpha for S-PHQ-9		0.79	0.81		

### Sensitivity

The sensitivity of S-PSS-10 questionnaire was assessed by comparing the mean scores across the three groups. As expected, the mean score was higher in the T2DM group compared to the two other groups (Table 5). Results of the ANOVA test suggested a significant difference in the mean scores between groups (*p* < 0.00). However, the findings of the *Post hoc* test for multiple comparisons (Bonferroni test) indicated that there was no significant difference between the mean scores of the ASMHC and HCC groups (*p* < 0.07) while the mean scores of the T2DM showed significant differences with the two other groups. Anyhow, the independent t-test results were significant between each group at (*p* < 0.05).

**Table 5:** Results of the multiple comparisons of the mean scores.

Test	Group	Groups	Mean difference	Std. Error	Sig.
*Post hoc* Bonferroni	T2DM	ASMHC	5.92^*^	0.69	0.00
		HCC	8.01^*^	0.78	0.00
	ASMHC	T2DM	−5.92^*^	0.69	0.00
		HCC	2.08	0.93	0.07
	HCC	T2DM	−8.01^*^	0.78	0.00
		ASMHC	−2.08	0.93	0.07
Independent *t*-test	T2DM	ASMHC	5.92	0.69	0.00
	T2DM	HCC	8.01	0.79	0.00
	ASMHC	HCC	−2.08	0.94	0.02

*Significant mean differences of the Bonferroni post hoc test (*p* < 0.05).

### Construct validity

#### Exploratory factor analysis

**Figure 2:**
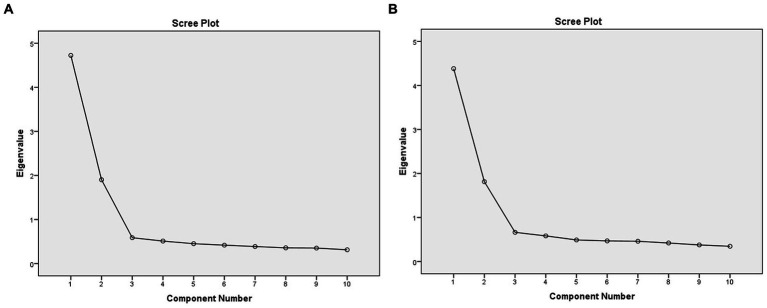
Scree plot analysis of the **(A)** total groups and **(B)** T2DM group.

The EFA results showed compelling evidence of adequacy for conducting the Factor Analysis in the Total, T2DM and ASMHC groups. All values resulted in the Correlation matrix (data not shown) were less than 0.6 and confirmed that there are no similar items in the questionnaire. The Kaiser-Meyer-Olkin (KMO) Measure of Sampling Adequacy was reported as 0.88, representing good sampling adequacy in the T2DM group. Also, the approximate Chi-square value of the Bartlett’s test of sphericity was 1274.8 and statistically significant (*p* < 0.000). Two factors were identified for the S-PSS-10 scale with eigenvalues greater than 1.0, accounting for 61.95% of the total variance. Factor 1 comprised 6 items representing “Perceived distress/negative feelings” (Items 1, 2, 3, 6, 9, and 10) with a variance of 43.81%, whereas Factor 2 consisted of 4 items representing “perceived distress/positive feelings” (Items 4, 5, 7, and 8) resulting a variance of 18.13%. All groups showed good compliance to the two-factor model (See Table 6). For combined groups, the total scree plot is presented in Figure 2.

**Table 6:** Results of the exploratory factor analysis.

Description	Total	T2DM	ASMHC	HCC
KMO	0.90	0.88	0.83	0.79
Chi-square value	2393.5	1274.8	539.0	276.0
Bartlett’s test significance	0.00	0.00	0.00	0.00
No of factors with eigenvalues >1	2	2	2	2
Variance: perceived distress	47.2%	43.8%	41.4%	38.3%
Variance: perceived coping	19.0%	18.1%	27.0%	21.0%

Also, there were two factors identified in the Rotated Component Matrix in the total and in the T2DM group. The factor loadings for the items ranged from 0.71 to 0.83. The respective factor loadings for the S-PSS-10 items are presented in Table 7.

**Table 7:** Rotated component matrix of the T2DM sample.

PSS-10 item questions	Factor 1		Factor 2		T2DM	Total	T2DM	Total
1. In the last month, how often have you been upset because of something that happened unexpectedly?	**0.77**	**0.83**	0.17	0.13
2. In the last month, how often have you felt that you were unable to control the important things in your life?	**0.79**	**0.80**	0.13	0.18
3. In the last month, how often have you felt nervous and “stressed”?	**0.79**	**0.81**	0.13	0.13
4. In the last month, how often have you felt confident about your ability to handle your personal problems?	0.19	0.19	**0.81**	**0.83**
5. In the last month, how often have you felt that things were going your way?	0.18	0.23	**0.78**	**0.80**
6. In the last month, how often have you found that you could not cope with all the things that you had to do?	**0.78**	**0.72**	0.17	0.23
7. In the last month, how often have you been able to control irritations in your life?	0.10	0.07	**0.72**	**0.75**
8. In the last month, how often have you felt that you were on top of things?	0.12	0.16	**0.77**	**0.81**
9. In the last month, how often have you been angered because of things that were outside your control?	**0.74**	**0.79**	0.09	0.09
10. In the last month, how often have you felt difficulties were piling up so high that you could not overcome them?	**0.71**	**0.77**	0.17	0.17

Extraction method: Principal Component Analysis; Rotation method: Varimax with Kaiser Normalization. Bold values represent the factor loadings above 0.5.

#### Confirmatory factor analysis

**Figure 3:**
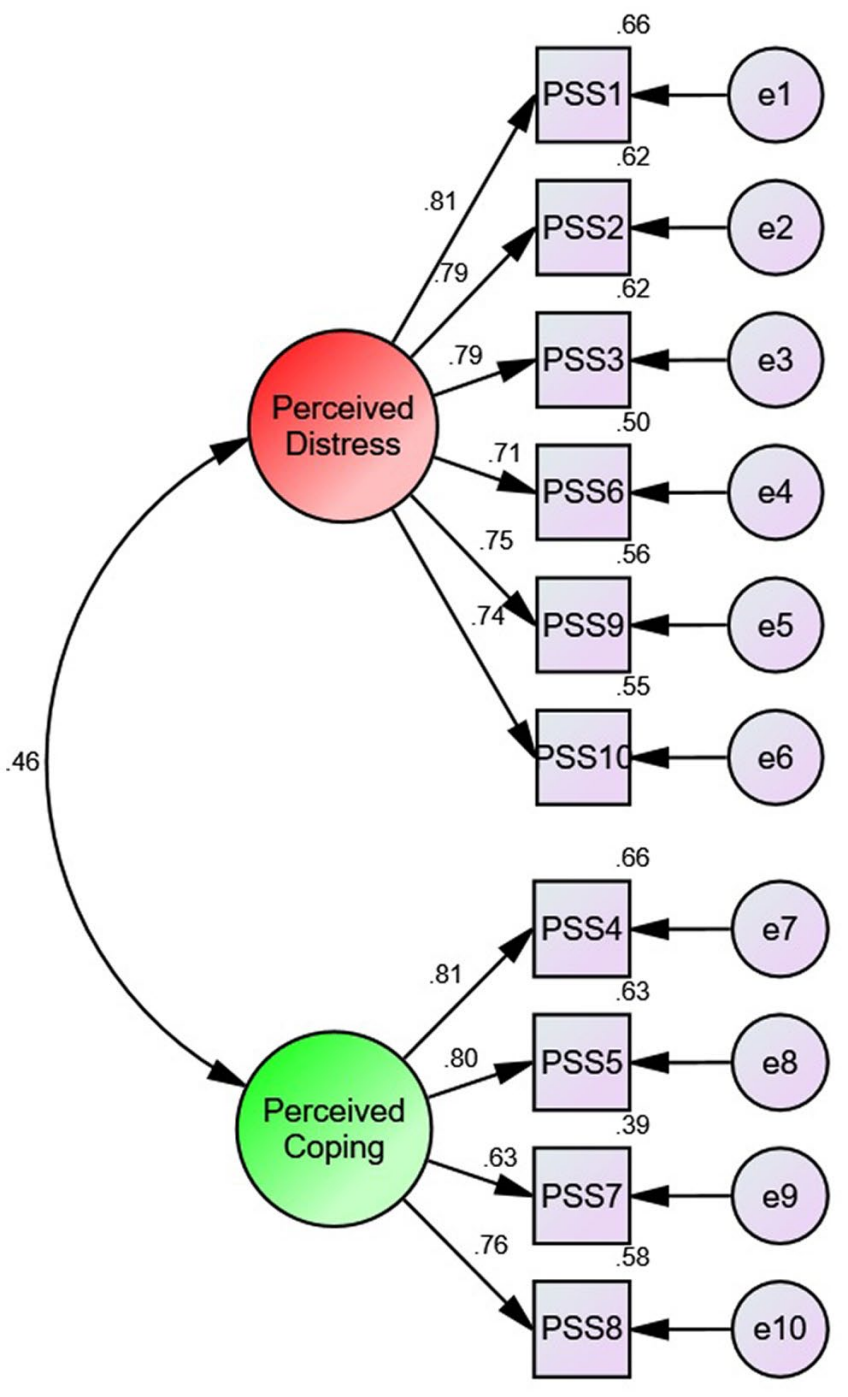
Path diagram with standardized estimates of the two factor Sinhalese PSS-10 items.

The CFA was performed to determine the goodness-of-fit for the two-factor model resulted from the EFA. An acceptable normality level was reported in the T2DM group, resulting in multivariate kurtosis values of less than 5. The maximum-likelihood estimation (MLE) method was employed to test the covariance matrix to assess the model fit of the sample data. All critical ratios under regression weights were above 1.96 and the results of the estimates of the regression weights were significant (data not shown). Considering the data of the total sample, the minimum and maximum standardized regression weights were reported as 0.71 and 0.81 (perceived distress/negative feelings) and 0.63 and 0.81 (perceived ping/positive feelings) while their squared loadings were ranged from 0.50 to 0.66 and 0.63–0.81, respectively. The Covariance between the perceived distress and perceived coping was recorded as 0.46 in the standardized estimate for the total groups. The path diagram of the total groups with the standardized estimates is presented in Figure 3.

The findings of the goodness-of-fit measures demonstrated that two factor model was adequate for all three groups, including the combined total sample group (see Table 8). In the T2DM group, the model fit measures were reported as Chi-square – χ^2^ (CMIN) = 32.14, degrees of freedom = 34, and a non-significant Likelihood ratio *p*-value of 0.559. Similarly, other model fit measures demonstrated good evidence for the two-factor model: CMIN/DF = 0.94; Root Mean square Error of Approximation (RMSEA) = 0.00; Normed Fit Index (NFI) = 0.97; Comparative Fit Index (CFI) = 1.0 and Tucker-Lewis index (TLI) = 1.0. The lowest likelihood ratio *p*-value of 0.056 was obtained in the ASMHC group; all values were non-significant indicating an excellent model fit. The CMIN/DF values ranged from 0.94 to 1.41 with a reasonable model fit. All RMSEA values were less than 0.08 and showed reasonable model fit. The NFI and values were in between 0.87 and 1.0 eliciting a reasonable fit while all CFI values were above 0.9 elucidating an excellent model fit.

**Table 8:** Model fit measures of the three groups.

Description	Total	T2DM	ASMHC	HCC	Threshold	Interpretation of the model fit
Absolute fit measurement						
Chi-square – χ^2^ (CMIN)	47.08	32.14	47.98	38.10	–	–
Degrees of freedom (DF)	34	34	34	34	–	–
Likelihood ratio *p*-value	0.06	0.55	0.05	0.28	>0.05	Exact fit
CMIN/DF	1.38	0.94	1.41	1.12	Between 1 and 3	Reasonable fit
RMSEA	0.02	0.00	0.06	0.04	<0.08	Reasonable fit
Relative fit indices						
NFI (Normal fit index)	0.98	0.97	0.91	0.87	>0.9	Reasonable fit
CFI (comparative fit index)	0.99	1.0	0.97	0.98	>0.95	Exact fit
TLI (Tucker-Lewis index)	0.99	1.0	0.96	0.97	>0.9	Reasonable fit

#### Concurrent validity

The Pearson correlation coefficient between the Sinhalese PSS-10 and validated Sinhalese PHQ-9 was reported as 0.64 in the T2DM group and 0.52 in the ASMHC group. As expected, S-PHQ-9 scale showed a significant positive correlation with the S-PSS-10 in both samples (*p* < 0.05).

## Discussion

The current study is the first attempt to evaluate the reliability and validity of the Sinhalese version of the PSS-10 scale in Sri Lanka. The analyses revealed that the Sinhalese-translated PSS-10 two factor questionnaires had substantial evidence for the elicitation of psychometric properties. Considering the internal consistency of total group, the Cronbach alpha values were similar to the previously published research in Germany (Bastianon et al., 2020). The reliability coefficients of the three groups ranged from 0.79 to 0.85 and were also the consistent with the studies conducted in Mexico and South Korea (Lee et al., 2015; Makhubela, 2022). The correlation coefficients of the test–retests were higher than 0.7 for T2DM and ASMHC and were significant. Though both values were significant, the ASMHC had a higher correlation coefficient than T2DM. A possible explanation is the shorter retesting time of the ASMHC group, which negated the effects of any events that might have affected the scores of the second interview resulting in increased reliability (Chaaya et al., 2010).

According to the literature, the sensitivity of the PSS-10 is comparatively higher among the people with chronic illnesses (Zhao et al., 2018; Soria-Reyes et al., 2023). Cohen et al. (1983) also reported that higher level of perceived stress is associated whenever there are failures among diabetics to control blood sugar levels (Wiernik et al., 2016; Hackett and Steptoe, 2017). As predicted, the mean perceived stress score of the T2DM sample was reported significantly higher compared with the other two groups eliciting a good sensitivity of the S-PSS-10. The mean perceived stress score of T2DM sample was obtained as 23.8 ± 6.0 and the value was comparable with previously published perceived stress scores of T2DM patients in Greece (Koloverou et al., 2014). Hence, the results obtained in our sample also supported the evidence for the validity among T2DM patients (Hanson and Pichert, 1986).

In principal component analysis, there were two factors identified which resulted eigenvalues greater 1. According to the previously published literature, these two factors have been identified and named as ‘perceived distress or perceived helplessness’ and ‘perceived coping or perceived self-efficacy.’ Furthermore, the findings of the Rotated component matrix for the total groups resulted in two identified factors which were similar to the previously published research in China where the values of the factor loadings ranged from 0.72 to 0.83 (Wang et al., 2011).

The likelihood of estimation showed strong evidence of validity during the CFA analysis. The likelihood ratio p-values of all three groups were not significant (*p* < 0.05). Assuming that the default models are correct, the probability of getting discrepancies as large as 47.08 and 32.149 are 0.55 and 0.06 in the total sample and T2DM group, respectively. Similar non-significant Chi-square values of likelihood estimation were also reported in the studies conducted in China and UAE (Chaaya et al., 2010; Wang et al., 2011). All RMSEA values were less than 0.08 and indicated a reasonable error of approximation with a comparable fit of the model in relation to the degrees of freedom. The relative fit indices of Bentler-Bonett normed fit index (NFI) and Tucker-Lewis index (TLI) values were between 0.9 and 1.0. According to their experience, models with overall fit indices around 0.9 can usually be improved substantially while TLI values close to 1 indicate a good model fit. The findings of the Bangladesh validity study have also resulted in similar findings with RMSEA = 0.05, CFI = 0.95 and TLI = 0.93 demonstrating a good validity in our sample groups (Mozumder, 2022). As predicted, the S-PSS-10 had positive associations with the S-PHQ-9 that was previously validated in Sri Lanka (Hanwella et al., 2014). This significant correlation in the T2DM group (*r* = 0.61) could be due to higher stress levels in T2DM patients owing to multiple comorbidities and complications as well as a higher tendency to have depression due to the chronicity of the illness (Carey et al., 1991; Gillani, 2011). Despite the two-factor structure of PSS-10, the PHQ-9 which has one-factor structure and PSS-10 were correlated significantly (*r* = 0.63) in previously published research supporting our evidence (López-Guerra et al., 2022). A brief summary of PSS-10 validity and reliability studies is presented in Table 1 to compare and contrast the findings of our study. All studies included for comparison were comprised of PSS-10 validated studies. Validity studies published with PSS-4 and PSS-14 were excluded as the current study employed the 10-item perceived stress scale.

Even though there are contradictory findings, the PSS-10 two factor structure has shown adequate psychometric evidence of validity. The diversity of construct validity results could be due to different statistical techniques, sample heterogeneity (i.e., age, sex, ethnicity, and culture), participant characteristics (i.e., diseased, community-based and specific groups) and sample size (Lee and Jeong, 2019). However, the factor analysis statistical techniques to assess the construct validity can be utilized effectively to provide the evidence of validity. Considering the findings of the previously validated studies, the current study has shown conclusive evidence for the validity of S-PSS-10.

Therefore, findings of this study have shown conclusive evidence for the validity of S-PSS-10. The two-factor structure of the S-PSS-10 possessed high internal consistency and reliability compared to the previously published research while showing strong evidence of concurrent validity. Essentially, the EFA and CFA analysis have provided acceptable model fit the evidence of the S-PSS-10 version. Hence, future research based in communities, clinical settings and busy outpatient clinics can use this validated questionnaire to assess perceived stress. Further, using the impact event scale or similar psychometric methods along with the S-PSS-10 could identify the specific life stressors contributing to perceived stress.

### Strengths and limitations

This study was conducted during the post-COVID period where the majority of the Sri Lankans are facing an economic melt-down which had led to unprecedented inflation. This may have contributed to elevated stress levels but would have affected all three groups similarly as it was a problem common to all Sri Lankans. Hence, the stress assessment across the three groups is higher and comparable. Importantly, this tool was administered in an out-patient clinic and wards of the NHSL showing evidence that S-PSS-10 tool can be practically administered to assess stress in similar busy clinical settings. Further, the study populations consisted of different subsets representing several groups which supports the generalizability of findings. Though the T2DM group had consecutive sampling, the convenient sampling techniques employed in the ASMHC, and HCC groups could undermine the significance of the study in the national context. Additionally, concurrently assessment of the convergent validity using an impact event scale would have enhanced the validation process.

## Conclusion

This study has provided evidence of validity and reliability for the S-PSS-10 scale. In future, this tool can be used to assess perceived stress across different groups, provided, there is evidence for internal consistency and factor structure the respective populations. Furthermore, the S-PSS-10 being a concise and easy to use questionnaire which can be administered within a short time period is an appropriate tool to be used in busy Sri Lankan clinic settings. This property makes it a good tool to be used for patients with chronic illnesses and pain. Identifying the psychometric properties of the S-PSS-10 could also provide valuable evidence for the healthcare management to assess stress of people with non-communicable diseases and those facing difficult social circumstances. Therefore, it is proposed that the S-PSS-10 can be used as a robust tool to screen for stress and thereby suggest interventions to prevent adverse physical and mental health problems and mitigate the adverse impact of these on the country’s economy.

## Data availability statement

The original contributions presented in the study are included in the article/Supplementary material, further inquiries can be directed to the corresponding author.

## Ethics statement

The studies involving human participants were reviewed and approved by the Ethics Review Committee (ERC), Faculty of Medicine, University of Colombo Sri Lanka. The patients/participants provided their written informed consent to participate in this study. The ERC certificate reference numbers are EC-20-010 and EC-19-106. The study is also registered under the Sri Lanka clinical trials registry under the registration number SLCTR/2021/012.

## Author contributions

AD, IR, and BM: conceptualization. BM, PP, IR, and AD: methodology. PP, BM, PK, IR, AH, WK, AB, KK, GP, and AD: investigation. AD, IR, DS, and JP: proofreading. WD and DS: resources. BM, KK, and PP: data curation. BM and AH: writing—original draft preparation. BM, AD, DS, JP, and IR: writing and editing. BM and AD: visualization. DS, JP, AD, IR, PK, SY, WH, WD, and US: supervision. WD and DS: project administration. DS, WD, JP, AD, IR, SY, and WH: funding acquisition. All authors have read and agreed to the published version of the manuscript.

## Funding

This project and the article processing charge (APC) of this publication were funded by the World Bank under the “Development-Oriented Research” scheme of the “Accelerating Higher Education and Expansion (AHEAD)” project, Ministry of Education, Sri Lanka (AHEAD/DOR/STEM+HEMS No. 78).

## Conflict of interest

The authors declare that the research was conducted in the absence of any commercial or financial relationships that could be construed as a potential conflict of interest.

## Publisher’s note

All claims expressed in this article are solely those of the authors and do not necessarily represent those of their affiliated organizations, or those of the publisher, the editors and the reviewers. Any product that may be evaluated in this article, or claim that may be made by its manufacturer, is not guaranteed or endorsed by the publisher.

## Supplementary material

SUPPLEMENTARY TABLE S1 Raw data file.

SUPPLEMENTARY DATA SHEET S1 Sinhalese version of the ten-item Perceived Stress Scale.
