# Supplementary material for: Validity and reliability of the Sinhalese version of the perceived stress scale questionnaire among Sri Lankans
Source: Front Psychol. 2023 Jun 16;14:1152002. doi: 10.3389/fpsyg.2023.1152002 (PMC10313401; doi:10.3389/fpsyg.2023.1152002)
Supplement: SUPPLEMENTARY DATA SHEET S1 — Sinhalese version of the ten-item Perceived Stress Scale. [file Data_Sheet_1.pdf]

යහපැවැත්ම සඳහා මඟ: අභිරුචි කළ සංගීත චිකිත්සාවෙන් ඇති විය හැකි මානසික සහනය නිසා සිදුවිය හැකි ජෛව අණුක වෙනස් වීම් ගවේෂණය කිරීම

පර්යේෂණය: AHEAD/DOR/STEM/+HEMS No 78

ප්‍රත්‍යක්ෂ ආතති මැනුම් ප්‍රශ්නාවලිය

පහත ප්‍රශ්නාවලිය පසුගිය මාසය තුළ ඔබට ඇති වූ සිතිවිලි හා හැඟීම් පිළිබඳ ව විමසනු ඇත. පහත සෑම ප්‍රශ්නයක් සඳහා ම පිළිතුරු වශයෙන් ඔබට කෙතරම් වාර ගණනක් දැනුනා ද?/ හැඟුනේ ද? යන්න ඊට අදාළ අංකය වටා කවයක් ඇඳීමෙන් ලකුණු කරන්න.

නම: .... දිනය: ....

වයස: ....   ස්ත්‍රී/ පුරුෂ භාවය: ගැහැණු/ පිරිමි/ වෙනත්

0	1	2	3	4
කිසිවිටෙක නොමැත	ආසන්න වශයෙන් නොමැත	සමහර විට	කිහිප වරක්	බොහෝ වරක්

1.	ගිය මාසයේ නිතරම වාගේ ඔබට සිද්ධ වුණු ඔබ බලාපොරොත්තු නොවූ දේවල් නිසා කණගාටුවෙන් සිටියා ද?	0	1	2	3	4
2.	ගිය මාසයේ නිතරම වාගේ ඔබගේ ජීවිතයේ ඉතාමත් වැදගත් දේවල් ඔබට පාලනය කරගන්න බැරි වුණා කියා දැනුනා ද?	0	1	2	3	4
3.	ගිය මාසයේ නිතරම වගේ ඔබ මානසික ආතතියෙන් සිටියා ද?	0	1	2	3	4
4.	ගිය මාසයේ නිතරම වගේ ඔබ ඔබගේ ප්‍රශ්න විසඳගන්න පුළුවන් වුනා කියන ආත්ම විශ්වාසයෙන් සිටියා ද?	0	1	2	3	4
5.	ගිය මාසයේ නිතරම වගේ ඔබට කැමති විදියට වැඩ සිද්ධ වුණා ද?	0	1	2	3	4
6.	ගිය මාසයේ නිතරම වගේ ඔබගේ තිබ්බ වැඩ ප්‍රමාණය දරන්න බැරි තරම් කියා දැනුනා ද?	0	1	2	3	4
7.	ගිය මාසයේ නිතරම වගේ ඔබගේ අපහසු කරදරකාරී හැඟීම් පාලනය කරගත්තේ ද?	0	1	2	3	4
8.	ගිය මාසයේ නිතරම වගේ ඔබගේ දේවල් හොඳට සිදු කලා කියලා ඔබට දැනුනා ද?	0	1	2	3	4
9.	ගිය මාසයේ නිතරම වගේ ඔබට පාලනය කරගන්න බැරි වුණ දේවල් ගැන ඔබට කේන්ති ගියා ද?	0	1	2	3	4
10.	ගිය මාසයේ නිතරම වගේ ඔබට විසඳගන්න බැරි තරම් ප්‍රශ්න/ ගැටළු ගොඩ ගැසුණ බවක් ඔබට දැනුනා ද?	0	1	2	3	4

මෙම ව්‍යාපෘතිය කොළඹ විශ්ව විද්‍යාලයේ වෛද්‍ය පීඨයේ ආචාරධර්ම සමාලෝචන කමිටුව විසින් අනුමත කර ඇත. 0112695300 (extension 240) (උදේ 9 සිට සවස 4 දක්වා) අමතන්න. විද්‍යුත් තැපෑලක් එවීමෙන් ද ඔබට පැහැදිලි කිරීම් ලබා ගැනීමට/ යම් ගැටළු වාර්තා කිරීමට හෝ අධ්‍යයනය පිළිබඳ පැමිණිලි කිරීමට හැක. අවශ්‍ය නම් කමිටුව අමතන්න.
